# Differences in all-cause mortality risk associated with animal and plant dietary protein sources consumption

**DOI:** 10.1038/s41598-023-30455-9

**Published:** 2023-02-28

**Authors:** Fahimeh Haghighatdoost, Noushin Mohammadifard, Parisa Zakeri, Jamshid Najafian, Masoumeh Sadeghi, Hamidreza Roohafza, Nizal Sarrafzadegan

**Affiliations:** 1 Interventional Cardiology Research Center, Cardiovascular Research Institute, Isfahan University of Medical Sciences, Isfahan, Iran; 2 Isfahan Cardiovascular Research Center, Cardiovascular Research Institute, Isfahan University of Medical Sciences, Isfahan, Iran; 3 Hypertension Research Center, Cardiovascular Research Institute, Isfahan University of Medical Sciences, Isfahan, Iran; 4 Cardiac Rehabilitation Research Center, Cardiovascular Research Institute, Isfahan University of Medical Sciences, Isfahan, Iran; 5 Heart Failure Research Center, Cardiovascular Research Institute, Isfahan University of Medical Sciences, Isfahan, Iran; 6 School of Population and Public Health, Faculty of Medicine, University of British Columbia, Vancouver, Canada

**Keywords:** Nutrition, Public health

## Abstract

The relationship between protein intake and mortality is still controversial. We prospectively examined the associations of dietary protein sources with all-cause mortality risk in the Isfahan cohort study (ICS). A total of 5431 participants, aged ≥ 35 years, were enrolled in the ICS, in 2001 and followed through 2013. The frequency of protein intakes from different sources was estimated through a validated food frequency questionnaire at baseline. Any new case of death was recorded over the follow-up duration. Hazard ratio (HR)s and 95% confidence interval (CI)s were estimated through Cox proportional hazards regression models. During a median follow-up of 11.3 years, 483 deaths were documented. Higher intakes of plant proteins (HR = 0.64, 95% CI 0.46, 0.91) and animal proteins (HR = 1.52, 95% CI 1.13, 2.05) were associated with a decreased and increased risk of mortality, respectively. Additional adjustment for some mediators did not considerably affect the associations for animal protein (HR = 1.55, 95% CI 1.15, 2.09), whereas led to a tendency towards lower risk for plant protein in the top quintile compared with the bottom one (HR = 0.67, 95% CI 0.48, 0.95; P trend = 0.06). Among specific major sources, higher intakes of nuts and fish were associated with a 27% (95% CI 0.58, 0.93) and 21% (95% CI 0.62, 1.01) lower risk of mortality, respectively. The inverse association between plant protein and mortality risk might be mediated by some metabolic disorders. However, our results suggest an independent positive association for animal protein and all-cause mortality.

## Introduction

Seventy percent of deaths occurred in world in 2013 have been attributed to chronic diseases^1^ which can be delayed by lifestyle modifications (i.e. dietary behaviors), even in the absence of any direct and clear biological mechanism. To date, many studies have investigated the effect of protein amount on cardiometabolic health^2^; however, it seems that protein type is matter much more than the quantity of protein^3^, given that they are consumed along with specific clusters of macronutrients, micronutrients, antioxidant, and phytochemical and convey different amino acids.

A large body of evidence supports the benefits of plant proteins in reducing risk of chronic diseases^4–8^, whereas red meat and processed meat may contribute to increased risk of various metabolic disorders^9^. Moreover, several prospective cohort studies have examined dietary protein sources in relation to mortality risk^9–16^. Although findings regarding nuts are mostly consistent and suggested an inverse association^17^, results for legumes, and animal sources of protein are less conclusive^18–20^. For example, legumes in most studies were not associated with mortality^21,22^, and a meta-analysis suggested just a slight decrease which was dependent on follow-up duration, sample size and geographic region^19^. Regarding red meat-mortality relation, while a meta-analysis suggested a region-specific association^20^, another one demonstrated very small inverse with low certainty association^18^. Likewise, in a recent meta-analysis^23^, Jayedi et al. showed a marginal inverse association; however, in their subgroup analysis according to the region of study, the association was only significant in Asian countries, but not Westerns. In contrast, higher fish consumption was not associated with the risk of mortality among Iranians in a 11-year follow-up study^14^.

The association of dietary protein sources with mortality may be influenced by several factors such as lifestyle, dietary pattern, and the contribution of protein sources to daily energy and protein intake. Nevertheless, to our knowledge, only a few studies have investigated these associations in developing countries, where the average per capita meat consumption is considerably lower than developed countries (25 vs. 88 kg/year)^24^. Therefore, we aim to further investigate the associations of dietary protein sources, including meat, fish, poultry, egg, dairy products, nuts and legumes, with all-cause mortality, using data from Isfahan cohort study (ICS).

## Methods and materials

### Study population

The ICS is an ongoing population-based longitudinal cohort study^25^, established in 2001 and includes 6504 adults (3168 men and 3336 women) aged ≥ 35 years. The ICS was conducted in three districts of central Iran and participants were recruited using stratified cluster random sampling method (2153 from Isfahan, 1028 from Najaf-Abad, and 3323 from Arak). Further detailed description about study design has been presented elsewhere^25^. At baseline, information about lifestyle factors, including dietary intake, was collected using face-to-face interview and participants were followed up biannually for outcomes of interest. In the absence of any cardiovascular event as the primary outcomes of ICS in biannual evaluations, all variables including lifestyle factors were assessed in the next survey after six years of follow-up (2007 and 2013). For our analysis, data from 5584 participants who attended for repeated measurements in both 2007 and 2013, and had complete information on dietary intake, and covariates were included. All participants provided a written informed consent. This study was performed in accordance with the declaration of Helsinki and approved by the Ethics Committee of the Research Council of Isfahan Cardiovascular Research Center, a World Health Organization collaborating center in Isfahan, Iran.

### Data collection

At enrolment, trained health professionals completed a general questionnaire about demographic, socioeconomic variables, lifestyle factors including dietary intakes, smoking status (current, former, or never), and physical activity, and medical history (e.g. the history of dyslipidemia, diabetes mellitus, hypertension, and medicine use) through a 30-min home interview^26,27^. Physical activity was assessed using a validated questionnaire^28^. Height and weight were measured using standard protocols, and body mass index (BMI) was measured by dividing weight in kg by height in meters squared.

### Dietary assessment

The habitual dietary intake of participants over the year preceding baseline, 2007 and 2013 was assessed using a validated 48-item food frequency questionnaire (FFQ)^29,30^. FFQ was completed by trained health professionals through face-to-face interviews and participants were asked to report the mean frequency of consumption of each food item in an open-ended format (daily, weekly, or monthly). If one food item was never consumed or less than once a month, they chose “never/seldom” presenting “zero” for frequency of consumption. Our FFQ did not have information about portion sizes; however, our validation study of this FFQ showed that portion sizes varied less than frequencies of consumption for most food items^30^. Dietary intake was converted into the frequency of consumption per week. Dairy products intake included the frequency intake of milk, yogurt, and cheese. Red meat included all types of beef and lamb, poultry included chicken and turkey. For fish intake, we did not consider canned fish, because its high sodium content could possibly mislead the results.

The validity of FFQ was examined against a single 24-h recall and two food records and demonstrated significant correlation between the frequency of animal protein, plant protein, and dairy products obtained from FFQ and the mean intake values obtained from a dietary recall and records (Spearman’s rank correlation coefficient for: animal protein = 0.294; P = 0.007, plant protein = 0.480; P < 0.001, dairy products = 0.467; P < 0.001, nuts = 0.468; P < 0.001)^30^.

### Ascertainment of deaths

Totally 483 deaths occurred after 11.3 years of follow-up. Information on mortality was gathered based on verbal autopsy from surviving close family members using a structured primary interview in which the first question was ‘is he/she alive?”.

### Statistical analysis

To compare general characteristics of participants at recruitment, we categorized participants according to the quantiles of animal (including red meat, poultry, fish, egg, and dairy products), plant (nuts and seeds, and legumes and soy), and total (plant and animal) protein consumption. Continuous and categorical variables were compared using analysis of variance (ANOVA) and chi-square test across the quartiles of dietary protein sources, respectively. Continuous variables were reported as mean ± SD, and categorical variables were described as percent. Age-, sex-, and BMI-adjusted of dietary intakes across the quartiles of dietary protein sources were compared using analysis of covariance (ANCOVA) and expressed as mean ± SE.

Person-years of follow-up was calculated from recruitment until the date of death or last follow-up date (2013), which ever occurred first. Crude and multivariate-adjusted hazard ratios (HRs) and 95% CI for association between dietary protein sources and all-cause mortality were estimated using Cox proportional hazards regression. Model 1 was controlled for age at baseline and sex. Model 2 was additionally adjusted for education and lifestyle factors including smoking, physical activity and BMI. Fruit and vegetables were adjusted beside other confounders in model 3. The mediating effects of hypertension, dyslipidemia and diabetes mellitus were further controlled in model 4. In this study, we used the frequency of consumption and their range was small when various protein sources were separately analyzed. Therefore, to have enough participants in each category, analysis based on individual dietary protein sources was performed based on the tertile of consumption frequency.

## Results

During 11.3 years of follow-up with 57,122.7 person-years, 483 deaths were documented in the ICS. The frequency consumption of animal and plant proteins was on average 9.7 ± 5.1 and 4.6 ± 3.6 times/week, respectively. Table 1 displays general characteristics of participants at baseline across the quintiles of animal, plant and total proteins. Compared with participants who consumed lower amounts of any of the animal, plant or total protein sources, those with higher consumption were more likely to be younger, male, physically active, highly educated, and current smoker and to have lower BMI and waist circumference. In addition, diabetes mellitus (DM), hypertension (HTN) and dyslipidemia were less frequent in higher quintiles of all three animal, plant and total protein sources compared with the lowest quintile.

**Table 1:** General characteristics of participants at baseline according to quintiles of protein sources in the ICS.

	Animal protein				Plant protein				Total protein			
	Q1	Q3	Q5	P value^1^	Q1	Q3	Q5	P value^1^	Q1	Q3	Q5	P value^1^
Participants, n	1080	1007	1086	–	1366	968	1036		1081	1111	1089	–
Age (years)	54.0 ± 12.6	50.0 ± 11.3	49.4 ± 11.2	< 0.0001	54.5 ± 12.4	49.1 ± 10.6	48.1 ± 10.5	< 0.0001	54.9 ± 12.6	49.4 ± 10.9	48.5 ± 10.6	< 0.001
Male (%)	42.4	50.6	52.5	< 0.0001	45.3	52.2	51.5	0.001	41.6	50.8	53.4	< 0.001
BMI (kg/m^2^)	27.0 ± 4.6	26.6 ± 4.6	26.2 ± 4.2	< 0.0001	27.2 ± 4.5	26.8 ± 4.3	26.3 ± 4.3	< 0.0001	27.2 ± 4.6	26.8 ± 4.6	26.4 ± 4.4	0.001
Waist circumference (cm)	96.0 ± 12.8	94.7 ± 11.9	92.6 ± 12.1	< 0.0001	97.0 ± 12.0	94.8 ± 12.3	93.0 ± 12.0	< 0.0001	97.1 ± 12.3	94.7 ± 12.0	93.0 ± 12.3	< 0.001
Physical activity (MET.min/week)	773.6 ± 523.4	885.3 ± 538.7	941.3 ± 567.8	< 0.0001	766.8 ± 517.8	912.4 ± 541.2	988.0 ± 586.4	< 0.0001	749.3 ± 517.6	867.2 ± 496.1	974.9 ± 578.2	< 0.001
Current smokers (%)	13.9	16.5	18.5	0.009	14.6	16.1	18.3	0.005	12.4	17.9	18.7	< 0.001
Education (%)				< 0.0001				0.014				< 0.001
0–5 years	80.6	65.9	70.3		73.9	66.8	69.6		78.8	68.3	68.5	
6–12 years	25.6	26.6	23.0		20.9	25.5	23.8		16.7	23.7	25.1	
> 12 years	3.9	7.4	6.7		5.3	7.6	6.6		4.5	8.0	6.4	
Diabetes (%)	13.9	11.4	10.1	0.011	15.1	9.6	8.3	< 0.0001	15.1	9.2	9.2	< 0.001
Dyslipidemia (%)	88.5	87.3	84.3	0.008	89.8	88.1	83.1	< 0.0001	89.0	87.2	84.7	< 0.001
HTN (%)	37.5	28.1	24.6	< 0.0001	37.3	24.6	23.8	< 0.0001	38.6	27.9	22.6	< 0.001

*BMI* body mass index, *HTN* hypertension, *MET.min/week* metabolic equivalent. h/day.

^1^Derived from 1-factor ANOVA and chi-square test for continuous and categorical variables, respectively.

^2^Values are mean ± SD (all such values).

The age- and sex-adjusted frequencies of different food items by the categories of animal, plant and total proteins are illustrated in Table 2. Participants with higher consumption of protein from each source had higher intake of all food groups, including fruit and vegetables, cereals, red meat, processed meat, poultry, fish, white meat, high fat dairy products, egg, nuts and seeds, and legumes and soy. The one exception for these associations was poultry across three quintiles of plant protein, which did not significantly differ across quintiles.

**Table 2:** Dietary intakes of participants according to quintiles of protein sources in the ICS.

	Animal protein				Plant protein				Total protein			
	Q1	Q3	Q5	P value^1^	Q1	Q3	Q5	P value^1^	Q1	Q3	Q5	P value^1^
Fruit and vegetables	9.6 ± 0.3	12.8 ± 0.2	16.0 ± 0.2	< 0.0001	12.1 ± 0.2	12.9 ± 0.3	15.4 ± 0.2	< 0.0001	10.0 ± 0.3	12.9 ± 0.2	16.0 ± 0.2	< 0.001
Cereals	23.2 ± 0.3	23.2 ± 0.2	25.1 ± 0.2	< 0.0001	22.8 ± 0.2	23.7 ± 0.2	25.4 ± 0.2	< 0.0001	22.8 ± 0.2	23.6 ± 0.2	25.4 ± 0.2	< 0.001
Red meat	1.9 ± 0.1	4.3 ± 0.1	7.4 ± 0.1	< 0.0001	3.9 ± 0.1	4.6 ± 0.1	6.2 ± 0.1	< 0.0001	2.0 ± 0.1	4.6 ± 0.1	7.3 ± 0.1	< 0.001
Processed meat	0.18 ± 0.04	0.33 ± 0.03	0.89 ± 0.03	< 0.0001	0.36 ± 0.03	0.41 ± 0.04	0.68 ± 0.03	< 0.0001	0.21 ± 0.04	0.40 ± 0.03	0.85 ± 0.03	< 0.001
Poultry	0.7 ± 0.07	1.7 ± 0.06	2.6 ± 0.05	< 0.0001	1.8 ± 0.06	1.8 ± 0.06	1.9 ± 0.06	0.205	1.0 ± 0.07	1.8 ± 0.06	2.5 ± 0.05	< 0.001
Fish	0.13 ± 0.03	0.32 ± 0.03	0.62 ± 0.02	< 0.0001	0.39 ± 0.03	0.36 ± 0.03	0.47 ± 0.03	0.001	0.19 ± 0.03	0.35 ± 0.03	0.57 ± 0.02	< 0.001
White meat^2^	0.8 ± 0.08	2.0 ± 0.07	3.3 ± 0.06	< 0.0001	2.2 ± 0.07	2.2 ± 0.08	2.4 ± 0.08	0.017	1.1 ± 0.08	2.1 ± 0.07	3.0 ± 0.07	< 0.0001
High fat dairy	0.05 ± 0.07	0.3 ± 0.06	2.2 ± 0.06	< 0.0001	0.4 ± 0.06	0.8 ± 0.07	1.2 ± 0.06	< 0.0001	0.04 ± 0.07	0.4 ± 0.06	2.0 ± 0.06	< 0.001
Egg	1.2 ± 0.08	1.9 ± 0.07	3.9 ± 0.06	< 0.0001	1.9 ± 0.07	2.2 ± 0.08	2.9 ± 0.07	< 0.0001	1.2 ± 0.08	2.0 ± 0.07	3.7 ± 0.06	< 0.001
Nuts and seeds	0.6 ± 0.1	0.9 ± 0.1	1.8 ± 0.1	< 0.0001	0.2 ± 0.07	0.6 ± 0.07	3.4 ± 0.07	< 0.0001	0.3 ± 0.09	0.7 ± 0.07	2.7 ± 0.07	< 0.001
Legumes and soy	2.7 ± 0.1	3.3 ± 0.1	4.8 ± 0.1	< 0.0001	1.2 ± 0.06	3.2 ± 0.06	6.9 ± 0.06	< 0.0001	1.7 ± 0.09	3.0 ± 0.07	5.8 ± 0.07	< 0.001
Animal protein	4.1 ± 0.1	8.9 ± 0.1	17.6 ± 0.1	< 0.0001	8.8 ± 0.2	10.2 ± 0.2	13.5 ± 0.2	< 0.0001	4.7 ± 0.1	9.4 ± 0.1	17.0 ± 0.1	< 0.001
Plant protein	3.3 ± 0.1	4.2 ± 0.1	6.6 ± 0.1	< 0.0001	1.4 ± 0.06	3.8 ± 0.07	10.3 ± 0.07	< 0.0001	2.0 ± 0.1	3.7 ± 0.1	8.5 ± 0.1	< 0.001
Total protein	7.5 ± 0.2	13.2 ± 0.2	24.2 ± 0.1	< 0.0001	10.2 ± 0.2	14.1 ± 0.2	23.8 ± 0.2	< 0.0001	6.7 ± 0.1	13.2 ± 0.1	25.5 ± 0.1	< 0.001

Values are age- and sex-adjusted Mean ± SE of frequency consumption/week.

^1^Derived from ANCOVA.

^2^White meat includes poultry and fish.

Table 3 shows the HRs and 95% CIs for all-cause mortality across the quintiles of animal, plant and total protein. In the crude model, compared with the first quintile, HRs in the highest quintile were 0.78 (95% CI 0.59, 1.04; P = 0.011) for animal protein, 0.33 (95% CI 0.24, 0.64; P < 0.0001) for plant protein and 0.53 (95% CI 0.40, 0.71; P < 0.0001) for total protein. Adjustment for age and sex, and education beside lifestyle factors including smoking, BMI and physical activity weakened the associations for all three sources and led to a non-significant association for animal and total protein. However, further control for fruit and vegetables resulted in a significant increase in the mortality risk for animal proteins when comparing the top with the bottom quintile (HR = 1.52, 95% CI 1.13, 2.05; P = 0.03). Significant inverse association was also observed between plant protein and mortality risk even after additional adjustment for fruit and vegetables (HR = 0.64, 95% CI 0.46, 0.91; P = 0.03). In model 4, adjustment for some metabolic disorders as mediators, such as diabetes, hypertension and dyslipidemia, slightly increased the association for animal sources (HR = 1.55, 95% CI 1.15, 2.09; P = 0.02), but led to a tendency towards lower risk of mortality in participants in the top quintile of plant protein compared with the bottom one (HR = 0.67, 95% CI 0.48, 0.95; P = 0.06). The association remained no longer significant for total protein in all adjusted models and just moderately changed in different models (model 4, HR for fifth quintile = 1.22, 95% CI 0.89, 1.67; P = 0.23). When animal, plant and total protein were considered a continuous variable, the same results were obtained (Table 3).

**Table 3:** Multivariate-adjusted HRs (95% CIs) of death according to quintiles of protein sources in the ICS.

	Quintiles of the frequency of consumption					P trend	Continuous variable	P-value
	Q1	Q2	Q3	Q4	Q5			
Animal protein								
Cases, n	116/1084	102/1111	71/1009	73/1141	85/1086			
Median (IQR)	4.2 (3.0, 5.0)	6.9 (6.2, 7.4)	9.0 (8.4, 9.4)	11.2 (10.5, 12.0)	16.2 (14.5, 18.9)			
Crude model	1 (Reference)	0.84 (0.64, 1.09)	0.66 (0.49, 0.88)	0.62 (0.46, 0.83)	0.78 (0.59, 1.04)	0.01	0.98 (0.96, 1.00)	0.03
Model 1	1 (Reference)	1.14 (0.88, 1.49)	1.02 (0.76, 1.37)	1.06 (0.79, 1.42)	1.37 (1.03, 1.82)	0.10	1.02 (1.00, 1.03)	0.07
Model 2	1 (Reference)	1.19 (0.91, 1.55)	1.04 (0.77, 1.41)	1.08 (0.80, 1.46)	1.43 (1.07, 1.91)	0.07	1.02 (1.00, 1.04)	0.03
Model 3	1 (Reference)	1.22 (0.93, 1.59)	1.08 (0.80, 1.47)	1.13 (0.83, 1.52)	1.52 (1.13, 2.05)	0.03	1.02 (1.00, 1.04)	0.01
Model 4	1 (Reference)	1.21 (0.93, 1.59)	1.07 (0.79, 1.45)	1.16 (0.86, 1.60)	1.55 (1.15, 2.09)	0.02	1.03 (1.01, 1.04)	0.006
Plant protein								
Cases, n	173/1370	88/984	72/970	71/1071	43/1036			
Median (IQR)	1.5 (1.0, 2.0)	3.0 (2.5, 3.0)	3.0 (2.5, 3.0)	6.0 (5.0, 7.0)	9.0 (8.0, 11.0)			
Crude model	1 (Reference)	0.71 (0.55, 0.92)	0.59 (0.44, 0.77)	0.53 (0.40, 0.70)	0.33 (0.24, 0.64)	< 0.001		
Model 1	1 (Reference)	0.90 (0.69, 1.16)	0.93 (0.70, 1.23)	0.87 (0.66, 1.15)	0.61 (0.44, 0.86)	0.01	0.91 (0.88, 0.94)	< 0.001
Model 2	1 (Reference)	0.90 (0.70, 1.17)	0.96 (0.73, 1.27)	0.86 (0.65, 1.14)	0.63 (0.45, 0.89)	0.02	0.97 (0.94, 1.00)	0.04
Model 3	1 (Reference)	0.90 (0.70, 1.17)	0.97 (0.73, 1.28)	0.86 (0.65, 1.15)	0.64 (0.46, 0.91)	0.03	0.97 (0.94, 1.00)	0.04
Model 4	1 (Reference)	0.93 (0.72, 1.21)	1.01 (0.76, 1.33)	0.90 (0.67, 1.19)	0.67 (0.48, 0.95)	0.06	0.98 (0.95, 1.05)	0.13
Total protein								
Cases, n	135/1086	102/1074	65/1112	77/1070	68/1089			
Median (IQR)	6.9 (5.2, 7.8)	10.2 (9.4, 11.0)	13.0 (12.4, 14.0)	16.7 (15.7, 17.9)	23.2 (20.9, 27.4)			
Crude model	1 (Reference)	0.76 (0.59, 0.98)	0.47 (0.35, 0.63)	0.60 (0.46, 0.0.80)	0.53 (0.40, 0.71)	< 0.001	0.97 (0.95, 0.98)	< 0.001
Model 1	1 (Reference)	1.12 (0.86, 1.45)	0.82 (0.61, 1.11)	1.09 (0.82, 1.45)	1.07 (0.80, 1.45)	0.79	1.00 (0.99, 1.01)	0.80
Model 2	1 (Reference)	1.13 (0.87, 1.47)	0.84 (0.62, 1.13)	1.09 (0.82, 1.45)	1.12 (0.82, 1.51)	0.67	1.00 (0.99, 1.02)	0.63
Model 3	1 (Reference)	1.16 (0.89, 1.51)	0.86 (0.63, 1.16)	1.13 (0.84, 1.52)	1.19 (0.86, 1.63)	0.45	1.01 (0.99, 1.02)	0.39
Model 4	1 (Reference)	1.17 (0.90, 1.52)	0.89 (0.65, 1.20)	1.16 (0.86, 1.55)	1.22 (0.89, 1.67)	0.32	1.01 (0.99, 1.02)	0.23

Model 1: HRs are adjusted for age (continuous) and sex.

Model 2: Further control for education, smoking status, physical activity and body mass index was made.

Model 3: Additionally adjusted for fruit and vegetables.

Model 4: Additionally adjusted for hypertension, diabetes mellitus and dyslipidemia.

As shown in Table 4, in the crude model, a lower risk of 21% for red meat (95% CI 0.63, 0.99; P = 0.046), 58% for processed meat (95% CI 0.33, 0.53; P < 0.0001), 42% for fish (95% CI 0.46, 0.73; P < 0.0001), 24% for egg (95% CI 0.57, 1.00; P = 0.047), 63% for nuts and seeds (95% CI 0.30, 0.47; P < 0.0001) and 31% for legumes and soy (95% CI 0.56, 0.86; P < 0.0001) was seen in the top tertile compared with the first one. However, after adjustment for potential confounders, all significance disappeared except for nuts and seeds, which remained strongly correlated with lower risk of mortality (T3: HR = 0.72, 95% CI 0.56, 0.91 and T2: HR = 0.83, 95% CI 0.62, 1.11; P = 0.005). Additional adjustment for mediators did not considerably change these associations. However, the results changed for fish and led to a lower risk in the top tertile compared with the bottom one (HR = 0.79, 95% CI 0.62, 1.01; P = 0.04). Given that only high-fat dairy products were considered in our FFQ, just above 71% of participants did not consume them and therefore we were not able to examine the association for it separately across tertile. According to the median of high fat dairy products, 334 and 113 deaths occurred in the first and the second medians, respectively. In addition, those in the higher median, compared with those in the lower median, had no increased risk of death in any of the models (HR in crude model = 0.85, 95% CI 0.69, 1.05, HR in model adjusted for potential confounders = 0.94, 95% CI 0.76, 1.18, and HR in model additionally adjusted for mediators = 0.99, 95% CI 0.79, 1.24).

**Table 4:** Multivariate-adjusted HRs (95% CIs) of death according to tertiles of specific major proteins sources in the ICS.

	Tertiles of the frequency of consumption			P trend	Continuous variable	P-value
	T1	T2	T3			
Red meat						
Cases, n	153/1574	156/1988	138/1869			
Median (IQR)	2.0 (1.0, 2.0)	3.7 (3.0, 4.0)	7.5 (7.0, 7.7)			
Crude model	1 (Reference)	0.78 (0.63, 0.98)	0.79 (0.63, 0.99)	0.05	0.97 (0.94, 1.00)	0.07
Model 1	1 (Reference)	1.16 (0.92, 1.45)	1.18 (0.93, 1.50)	0.16	1.03 (0.99, 1.06)	0.11
Model 2	1 (Reference)	1.18 (0.94, 1.48)	1.20 (0.95, 1.52)	0.12	1.03 (1.0, 1.06)	0.07
Processed meat						
Cases, n	353/3303	5/150	89/1978			
Median (IQR)	0 (0.0, 0.0)	0.23 (0.23, 0.23)	1.0 (0.7, 1.3)			
Crude model	1 (Reference)	0.29 (0.12, 0.70)	0.42 (0.33, 0.53)	< 0.001	0.63 (0.52, 0.75)	< 0.001
Model 1	1 (Reference)	0.62 (0.25, 1.50)	0.92 (0.71, 1.17)	0.43	0.99 (0.87, 1.13)	0.88
Model 2	1 (Reference)	0.59 (0.24, 1.43)	0.91 (0.71, 1.17)	0.41	0.99 (0.86, 1.13)	0.84
Poultry						
Cases, n	105/1237	227/2823	115/1371			
Median (IQR)	0.2 (0.0, 0.5)	1.0 (1.0, 2.0)	3.0 (3.0, 5.0)			
Crude model	1 (Reference)	0.92 (0.73, 1.16)	0.94 (0.72, 1.23)	0.69	1.03 (0.98, 1.08)	0.22
Model 1	1 (Reference)	0.97 (0.77, 1.24)	1.00 (0.76, 1.33)	0.96	1.03 (0.98, 1.08)	0.27
Model 2	1 (Reference)	0.97 (0.76, 1.23)	0.97 (0.73, 1.28)	0.81	1.02 (0.97, 1.07)	0.52
Fish						
Cases, n	260/2521	87/1363	100/1547			
Median (IQR)	0 (0.0, 0.0)	0.23 (0.23, 0.23)	1.0 (0.7, 1.3)			
Crude model	1 (Reference)	0.58 (0.45, 0.73)	0.58 (0.46, 0.73)	< 0.001	0.93 (0.83, 1.05)	0.26
Model 1	1 (Reference)	0.80 (0.63, 1.03)	0.83 (0.65, 1.06)	0.09	1.04 (0.93, 1.15)	0.50
Model 2	1 (Reference)	0.79 (0.61, 1.01)	0.79 (0.62, 1.01)	0.04	1.02 (0.92, 1.14)	0.70
White meat						
Cases, n	172/1898	131/1686	144/1847			
Median (IQR)	0.5 (0.2, 1.0)	2.0 (1.2, 2.0)	4.0 (3.0, 5.0)			
Crude model	1 (Reference)	0.83 (0.66, 1.04)	0.82 (0.66, 1.02)	0.08	1.01 (0.97, 1.05)	0.58
Model 1	1 (Reference)	0.98 (0.78, 1.24)	0.97 (0.76, 1.22)	0.78	1.02 (0.98, 1.06)	0.24
Model 2	1 (Reference)	0.97 (0.77, 1.23)	0.92 (0.73, 1.17)	0.51	1.01 (0.97, 1.05)	0.50
Egg						
Cases, n	132/1426	92/1214	76/1160			
Median (IQR)	1.0 (0.0, 1.0)	2.0 (2.0, 2.0)	3.0 (3.0, 7.0)			
Crude model	1 (Reference)	0.84 (0.65, 1.10)	0.76 (0.57, 1.00)	0.05	1.003 (1.00, 1.005)	0.01
Model 1	1 (Reference)	1.06 (0.81, 1.39)	1.14 (0.85, 1.52)	0.39	1.001 (0.999, 1.003)	0.17
Model 2	1 (Reference)	1.11 (0.84, 1.45)	1.18 (0.89, 1.58)	0.24	1.001 (0.999, 1.003)	0.15
Nuts and seeds						
Cases, n	295/2519	57/876	95/2036			
Median (IQR)	0 (0.0, 0.0)	0.5 (0.2, 0.5)	2.0 (1.0, 3.0)			
Crude model	1 (Reference)	0.51 (0.38, 0.68)	0.37 (0.30, 0.47)	< 0.001	0.80 (0.74, 0.87)	< 0.001
Model 1	1 (Reference)	0.83 (0.62, 1.11)	0.72 (0.56, 0.91)	0.005	0.95 (0.89, 1.01)	0.12
Model 2	1 (Reference)	0.84 (0.63, 1.12)	0.73 (0.58, 0.93)	0.01	0.96 (0.90, 1.02)	0.18
Legumes & soy						
Cases, n	217/2078	97/1421	133/1932			
Median (IQR)	2.0 (1.0, 2.0)	3.0 (3.0, 3.0)	6.0 (4.0, 7.0)			
Crude model	1 (Reference)	0.64 (0.51, 0.82)	0.69 (0.56, 0.86)	< 0.001	0.93 (0.90, 0.97)	0.001
Model 1	1 (Reference)	0.83 (0.65, 1.05)	0.90 (0.72, 1.12)	0.28	0.98 (0.94, 1.01)	0.21
Model 2	1 (Reference)	0.85 (0.67, 1.09)	0.92 (0.73, 1.15)	0.40	0.98 (0.94, 1.02)	0.31

Model 1: HRs are adjusted for age (continuous), sex, education, smoking status, physical activity, body mass index and fruit and vegetables.

Model 2: Additionally adjusted for hypertension, diabetes mellitus and dyslipidemia.

Additional analysis revealed that after adjustment for potential confounders, each time increase in the frequency of various protein sources was not related to the risk of all-cause mortality (Table 4). The corresponding values for high-fat dairy products were: HR = 1.05, 95% CI 1.00, 1.10; P = 0.06.

## Discussion

In the current prospective cohort study of Iranians, after 11 years of follow-up, we observed a positive association for animal protein but an inverse association for plant protein sources with all-cause mortality, though the association between plant protein and mortality risk was mediated by some metabolic disorders. Total protein was not significantly related to the risk of all-cause mortality. Further analyses based on the type of protein from different sources revealed inverse associations between nuts and fish intake and mortality risk which were not influenced by various confounders and mediators.

To date, several epidemiological studies have investigated the association between dietary protein sources and mortality. Consistently, in studies which protein sources were examined as plant or animal, an inverse association for plant sources and a direct association for animal sources have been reported^15,31–33^. Nevertheless, when specific major proteins sources were separately investigated in relation to mortality risk, contradictory results have been found. For example, higher red meat intake was associated with increased risk of mortality in American adults, Finish men and Swedish men and women^16,33,34^, but not in the Netherlands Cohort Study and the Golestan Cohort Study in Iran^10,14^, like our findings. While poultry^10^, dairy products^35^ and egg^14^ were associated with lower risk of overall mortality in some studies, there are some studies which failed to find any significant association for these sources^33,36,37^ which is in concordance with ours. Similar discrepancy is also observed for fish. Although an earlier study among Iranians reported a lower risk for cancer death by higher fish consumption, they found no association for fish and all-cause mortality. Similarly, in Finnish men a null association was found^33^ but in the Netherlands Cohort Study^10^, a direct association was obseved. Furthermore, nuts and legumes have been associated with lower mortality risk in some studies^10,14^, but not all^21,22,38^.

The reasons underlying different association between protein sources and overall mortality might be related to their relation with diet quality^6^. Consuming less than 70% of total protein from animal sources was associated with greater healthy eating index score in young adults^39^. Additionally, each protein source is an exclusive package of nutrients. For instance, animal protein usually comes with saturated fatty acids and detrimental amino acids including branched chain and aromatic amino acids, but plant protein is accompanied by antioxidants, phytochemicals, polyunsaturated fatty acids, fiber, and a combination of beneficial amino acids like arginine, cysteine, glutamine/glutamate, and glycine^6^. Besides, protein foods preference, the foods served with them^6^, and other health-related factors can be affected by behavioral and sociodemographic factors^40,41^. For example, in France, higher fish consumption was associated with better sociodemographic status, more physical activity, less smoking and eating 3 meals a day more frequently^6^, and in the US eating fish was associated with lower intake of dairy foods and red meat but higher intakes of vegetables^42^.

Despite potential mechanisms underlying the positive link between red meat and mortality, i.e. higher saturated fatty acids and heme iron content, we found no significant association. This might be explained by the low average intake of red meat in Iran compared with Western countries which mostly reported a positive link^9,10,43^. Red meat may also interact with other dietary factors^34,44^. In a Swedish cohort study, when processed meat intake was < 20 g/days, unprocessed meat was not associated with the risk of mortality^44^. Moreover, although higher meat intake can increase the production of carcinogenic N-nitroso compounds^45^, fruit and vegetables, rich in antioxidants compound, restrain their harm effects^46^, and may potentially affect the final risk. Therefore, it is relevant to consider the confounding effect of these factors. However, despite a null association between various sources of animal proteins and mortality, we found a direct link for animal protein sources that might be owing to the combined effect of red meat and egg.

This study has several limitations. First, to assess dietary intakes, we used a FFQ without portion sizes which does not allow us to determine the exact amount of protein foods as well as energy intake. Therefore, it was not possible to us to control the confounding effect of energy but to deal with this issue, we adjusted BMI as surrogate. Second, despite using a validated FFQ in this study, measurement errors due to recall bias of habitual intake may occur which can attenuate associations^47^. Moreover, all the Spearman correlation coefficients are less than 0.5 in our study population. This means that observed associations are weaker and somewhat conservative than those achieved by true nutrient indices measured by gold standard methods, such as weighted food records or biomarkers^48^. Third, although we adjusted our results for various confounders, the confounding effect of residual or bias related to unknown or unmeasured factors cannot be completely ruled out. Fourth, adjustment for some mediating factors like metabolic disorders in the last model may be an over adjustment and underestimates the true association. Finally, alcohol consumption was not adjusted in the present study, however, since this study was conducted on a Muslim population. Its consumption cannot be concern. Besides, our study has its own strengths including its large sample size, longitudinal design and the high between-individual variety in various specific major proteins sources. In addition, data were collected from both rural and urban areas in three different counties which can, at least to some extent, reveal dietary diversity, especially those related to the frequency consumption of various dietary proteins sources, in this population. This, consequently, improve the generalizability of our findings to populations with similar characteristics to our population.

In summary, this prospective cohort study indicates an inverse association for fish and nuts with all-cause mortality even after control for potential confounders. In addition, despite a tendency toward lower risk of all-cause mortality in individuals with higher plant protein consumption after adjustment for metabolic disorders, the direct association for animal protein remained strongly significant. Further well designed randomized clinical trial studies are suggested to examine casualty effects of protein intake on death and confirm our findings.

## Data Availability

The datasets used and/or analysed during the current study available from the corresponding author on reasonable request.
